# 33764 Cost-effectiveness of Initial Treatment Strategies for Localized Prostate Cancer: A Systematic Review

**DOI:** 10.1017/cts.2021.734

**Published:** 2021-03-30

**Authors:** B. Malik Wahba, Tarik Phillips, Kenneth Sands, Judith Lieu, Alexander K. Chow, Nicholas Pickersgill, Michelle Doering, Eric H Kim

**Affiliations:** Washington University in St. Louis School of Medicine

## Abstract

ABSTRACT IMPACT: We compare the cost-effectiveness of treatments for early prostate cancer, and propose how to maximize the value of care within an increasingly cost-constrained healthcare climate. OBJECTIVES/GOALS: Each year 192,000 men in the United States are diagnosed with prostate cancer. With various treatment options available, there is a growing role for cost-effectiveness analyses which may help maximize the value of care to the patient. In this review we compare the cost-effectiveness of primary treatments for clinically localized prostate cancer. METHODS/STUDY POPULATION: In this systematic review we aim to compare the cost-effectiveness or cost-utility of primary treatment strategies for clinically localized prostate cancer. This review, which adheres to 2009 PRISMA guidelines, included studies of men with clinically localized prostate cancer comparing at least two treatment strategies using the incremental cost-effectiveness ratio (ICER). We included analyses only of the United States healthcare system with at least 10 years of follow-up. These studies were published from 2006 to 2019 and generally included men with low or low to intermediate risk prostate cancer. Most studies reported outcomes for men age 65-70. All studies were prospective simulated trials and used a Markov model to simulate patient outcomes. RESULTS/ANTICIPATED RESULTS: Ten articles were included in the analysis. All studies used a Markov model to simulate a randomized trial. Six studies primarily compared radiation modalities, and four compared observation with immediate treatment. There was substantial heterogeneity in treatment protocols and the patients being simulated. Sensitivity analyses showed these models to be influenced by utility values and length of follow-up. A meta-analysis was not possible as no studies reported the variance of the primary outcome. Heterogeneity in study design limited comparisons of treatments across studies. However, these models were sensitive to patient-specific clinical factors, including life expectancy and the utility during and after each treatment. DISCUSSION/SIGNIFICANCE OF FINDINGS: These studies indicate collectively that the cost-effectiveness of prostate cancer treatment for similarly staged men may be heavily impacted by comorbidities and personal preferences. As the US moves towards value-based care, patient preferences may continue to drive the preferred treatment for newly diagnosed prostate cancer.

